# Study of Menopausal Symptoms using Menopause Rating Scale at a Tertiary Care Center: A Descriptive Cross-sectional Study

**DOI:** 10.31729/jnma.5200

**Published:** 2020-10-31

**Authors:** Asmita Pandey, Chanda Karki, Veena Rani Shrivastava, Dipti Shrestha, Pratigyan Gautam

**Affiliations:** 1 Department of Obstetrics and Gynaecology, Kathmandu Medical College and Teaching Hospital, Sinamangal, Kathmandu, Nepal

**Keywords:** *menopause*, *quality of life*, *questionnaire*

## Abstract

**Introduction:**

Menopause is the permanent cessation of menstruation for more than a year resulting from the loss of follicular activity of the ovaries. It is manifested by vasomotor, psychological, and urogenital symptoms which can be assessed by an internationally accepted scale known as Menopause Rating Scale. This study was conducted to find out the issues of perimenopausal women and proceed for management and proper counseling.

**Methods:**

A descriptive cross-sectional study was conducted among women visiting the gynecological outpatient department of a tertiary care hospital from June 2017 to May 2018 using the Menopause Rating Scale. Ethical approval was taken from the Institutional Review Committee (reference number 20122016). Convenient sampling was done. Statistical Package for the Social Sciences version 20.0 was used for data analysis. Point estimate at 90% confidence interval was calculated along with frequency and proportion for binary data.

**Results:**

Out of 189 perimenopausal women interviewed, the mean age of menopause was found to be 50.2±2.1 years. The most common gynecological symptoms among the study population was abnormal uterine bleeding 66 (34.9%) followed by abnormal vaginal discharge 50 (26.5%). Among symptoms in Menopause Rating Scale, the depressive mood was found in 99 (52.4%) cases followed by joint and muscular discomfort 88 (46.6%) and bladder problems in 87 (46%). None of the women had a score on the Menopause Rating Scale more than 16 and did not require management for their problem.

**Conclusions:**

Most of the women didn't know menopausal symptoms. However, none required intervention from gynecologists for their problems reflecting better quality of life.

## INTRODUCTION

Menopause is the permanent cessation of menstruation for more than a year resulting from the loss of follicular activity of the ovaries. The average age of women experiencing their final menstrual period is 51.5 years. Several factors like, environmental, genetic, surgical influences, diet, exercise, and reproductive history is associated with the severity of symptoms of menopause.^1–3^ Symptoms can be assessed by an internationally accepted tool known as the Menopause

Rating scale. It is a health-related quality of life scale which measures the severity of aging symptoms and their impact on women's lives.^4^

Menopause, despite being a natural change, can be psychologically and physically challenging but is mostly ignored by women of countries like ours and we are lagging in tracing such women and giving them proper counseling and management.

This study was conducted to find out the severity of symptoms among peri- and post-menopausal women using the Menopause Rating Scale at a tertiary care center.

## METHODS

A descriptive cross-sectional study was conducted in the out-patient department of Obstetrics and Gynecology of Kathmandu Medical College and Public Limited, Sinamangal, Kathmandu in women of 40-60 years of age from June 2017 to May 2018. Ethical approval was taken from the Institutional Review Committee (IRC), Kathmandu Medical College Public Limited (reference number 20122016). All the participants were informed about the study, written consent was taken, and had options to withdraw from it anytime. Ladies with long-term histories of psychiatric illness such as depression, anxiety, schizophrenia, and those not giving consent were excluded from the study. Pretested, an internationally validated questionnaire was used for data collection. As the questionnaire is in English, questions were verbally asked each participant by the interviewer. The convenient sampling method was used.

Sample size calculation was done as,

$$\begin{array}{ll} \text{n} & \text{= }{\text{Z}}^{\text{2}}\times \text{p}\times \text{(1}-\text{p)}/{\text{e}}^{\text{2}}\\ & \text{= }1.64^{\text{2}}\times 0.8\times (1-0.8)/0.05^{\text{2}}\\ & \text{= }0.43/0.0025\\ & =172 \end{array}$$

Where,

n = sample size
p = prevalence, 80% taken from the previous study^8^
e = margin of error, 5%
Z = 1.64 at 90% confidence interval

Taking 10% non-response, the final sample size was 189.

Collected data were entered and Statistical Analysis was performed using Statistical Package for the Social Sciences (SPSS) version 20. Point estimate at 90% confidence interval was calculated along with frequency and proportion for binary data.

## RESULTS

**Figure 1:**
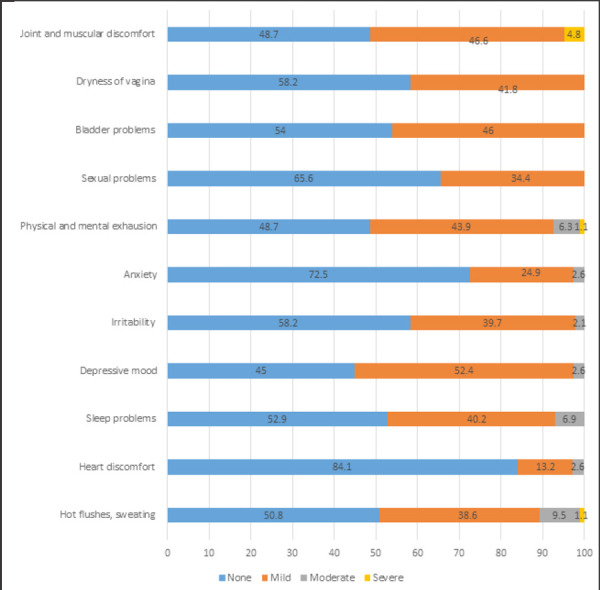
The severity of menopausal symptoms in the Menopause Rating Scale.

Out of 189 perimenopausal women, the mean age of menopause was found to be 50.2±2.1 at 90% Confidence Interval. The maximum number of women had primary education 89 (47.1 %) followed by lower secondary 47 (24.9 %). One hundred fifteen (60.8%) women were homemakers. Only 64 (33.9%) women knew about menopause. The most common symptoms in the Menopause Rating Scale were depressive mood 99 (52.4 %) in milder form followed by joint and muscular discomfort 88 (46.6 %), bladder problems 87 (46%). Physical and mental exhaustion was seen in 83 (43.9%). Most of the symptoms were mild. Irritability was seen in 75 (39.7 %). The severe symptom was hot flushes in 2 (1.1 %), physical and mental exhaustion in 2 (1.1 %), joint and muscular discomfort in 9 (4.8 %) (Figure 1).

None of the women had a score of more than 16 on the Menopause Rating Scale which means no one required management for their problem (Table 1).

**Table 1:** Grading of symptoms in Menopause Rating Scale.

Grading in Menopause Rating Scale	n (%)
<5	116 (61.4%)
>10	6 (3.2%)
5-10	67 (35.4%)
Total	189 (100%)

**Figure 2:**
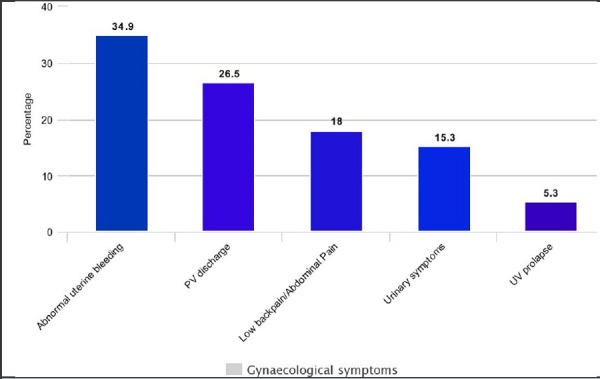
Gynecological symptoms among the study population.

The most common gynecological symptoms among women of perimenopausal age group were found to be abnormal uterine bleeding 66 (34.9%) followed by abnormal vaginal discharge 50 (26.5%) and low back pain with or without abdominal pain in 34 (18%). Urinary symptoms were seen in 29 (15.3%) and Uterovaginal prolapse in 10 (5.3 %)(Figure 2).

The effect of menopausal symptoms was assessed in the daily life of women. Work efficiency was hampered in 21 (11.1%) women in a milder form. Home responsibilities were affected in 17 (9%). Relationships with co-workers, family, and social life activities were hampered in only a few of the study population.

## DISCUSSION

Among 189 women of age group 40-60 enrolled in the study, 52.9 % of women were premenopausal and the rest were postmenopausal. The mean age of menopause was found to be 50.2 which is higher in comparison to other similar studies done in Nepal. The mean age previously described is between 47 to 50 years.^5–10^ Increase in age at menopause might be because of the improved lifestyle of women and change in the dietary habit in recent years known to delay menopause.^11^

Only 33.9% of women knew about menopause in this study and none were using Hormone replacement therapy though the study was conducted in an urban part of the country. Whereas Paudel et al. found 1.8% of women were using Hormone replacement therapy.^9^

The most common symptom in the Menopause Rating Scale was the depressive mood in 52.4 % in mild form and second, being joint and muscular discomfort 46.6%. In contrast to a study done by Ghimire et al. where the sexual problem was the most common one followed by joint and muscular discomfort 9.5%.^5^ Their study was done in rural Nepal whereas our study was done inthe urban part of the country. This might have been the region behind different results. In a study conducted by Rajbhandari et al. loss of sexual interest was the most common symptom that is 95.3% followed by joint and body pain in 31.3%.^6^

This study has a consistent result with the study done by Gyawali et al. who found joint and muscular discomfort as the most common symptom.^7^ Hot flushes is the commonest symptom in most of the studies conducted outside Nepal. In the current study, 50.8% of women did not complain of a hot flush. 38.6% complained of a milder form of a hot flush, moderate in 9.5%, and severe in 1.1%.

Heart discomfort was not a prevalent symptom in the current study. It was seen in a milder form in 13.2% cases and moderate in 2.6% whereas, in a study done by Marahatta, palpitation was seen in more cases that is 53%.^8^ Sleeping disturbance was seen more commonly in women of the perimenopausal age group in a study done by Marahatta RK 84 % and Singh A, Pradhan SK 62.7% but in this study, it was seen only in 40.2% of cases in a milder form.^8,12^

Bladder problems were seen in 46% of women in a milder form in the current study which was consistent with the study done by Ahmed et al. 37.7 % and in contrast to the study done by Rahman et al. 12.8%.^13,14^

In a study done by Mazhar SB, Rasheed S, the Menopause Rating Scale ranged from 9-21 score with a mean of 12.^15^ In our study most common ranking was below 5 that is 61.4%, 5-10 was seen in 35.4% and more than 10 was in 3.2 % cases. None of them had a score of more than 16, hence none required intervention from gynecologists in the current study.

The limitation of this study is the type of study that is the study design. More longitudinal studies could be conducted in the future as they would be more useful in assessing the menopausal symptoms and can be done in a larger area representing women from different parts of the country. The study population is hospital-based and therefore sample size might not be representative to give a generalization for the entire population.

## CONCLUSIONS

The purpose of this study was to study about menopausal symptoms among perimenopausal women using the Menopause Rating Scale and to find out the age of menopause. The age at menopause was found to be higher than in other similar studies, it might be because of the improved lifestyle of women and change in the dietary habit in recent years. Most of the women did not know about menopause and none knew the management options for menopausal symptoms. This focuses on the need for counseling for women in this age group. This would help a woman cope with the symptoms and prepare her mentally for the climacteric phase. Many women hesitate to talk about it which is lagging us behind in managing the symptoms. This is a phase in every woman's life and she has the rights to live her life peacefully which demands more such studies in both rural and urban parts of the nation along with counseling programs.
